# Site-Dependent Lineage Preference of Adipose Stem Cells

**DOI:** 10.3389/fcell.2020.00237

**Published:** 2020-04-15

**Authors:** Tingliang Wang, Ryan C. Hill, Monika Dzieciatkowska, Lian Zhu, Aniello M. Infante, Gangqing Hu, Kirk C. Hansen, Ming Pei

**Affiliations:** ^1^Stem Cell and Tissue Engineering Laboratory, Department of Orthopedics, West Virginia University, Morgantown, WV, United States; ^2^Department of Plastic and Reconstructive Surgery, Shanghai Ninth People’s Hospital Affiliated with Shanghai Jiao Tong University School of Medicine, Shanghai, China; ^3^Department of Biochemistry and Molecular Genetics, University of Colorado Denver, Aurora, CO, United States; ^4^Bioinformatics Core Facility, West Virginia University, Morgantown, WV, United States; ^5^Department of Microbiology, Immunology and Cell Biology, School of Medicine, West Virginia University, Morgantown, WV, United States; ^6^WVU Cancer Institute, Robert C. Byrd Health Sciences Center, West Virginia University, Morgantown, WV, United States

**Keywords:** adipogenesis, adipose stem cell, chondrogenesis, infrapatellar fat pad, osteogenesis, subcutaneous adipose tissue

## Abstract

Adult stem cells have unique properties in both proliferation and differentiation preference. In this study, we hypothesized that adipose stem cells have a depot-dependent lineage preference. Four rabbits were used to provide donor-matched adipose stem cells from either subcutaneous adipose tissue (ScAT) or infrapatellar fat pad (IPFP). Proliferation and multi-lineage differentiation were evaluated in adipose stem cells from donor-matched ScAT and IPFP. RNA sequencing (RNA-seq) and proteomics were conducted to uncover potential molecular discrepancy in adipose stem cells and their corresponding matrix microenvironments. We found that stem cells from ScAT exhibited significantly higher proliferation and adipogenic capacity compared to those from donor-matched IPFP while stem cells from IPFP displayed significantly higher chondrogenic potential compared to those from donor-matched ScAT. Our findings are strongly endorsed by supportive data from transcriptome and proteomics analyses, indicating a site-dependent lineage preference of adipose stem cells.

## Graphical Abstract

Lineage preference of adult stem cells might be influenced by local matrix microenvironment. In this study, stem cells from subcutaneous adipose tissue (ScAT) had more adipogenic capacity and those from infrapatellar fat pad (IPFP) possessed advantageous chondrogenic potential; these findings are fully endorsed by molecular evidence from RNA Seq and proteomic analyses.

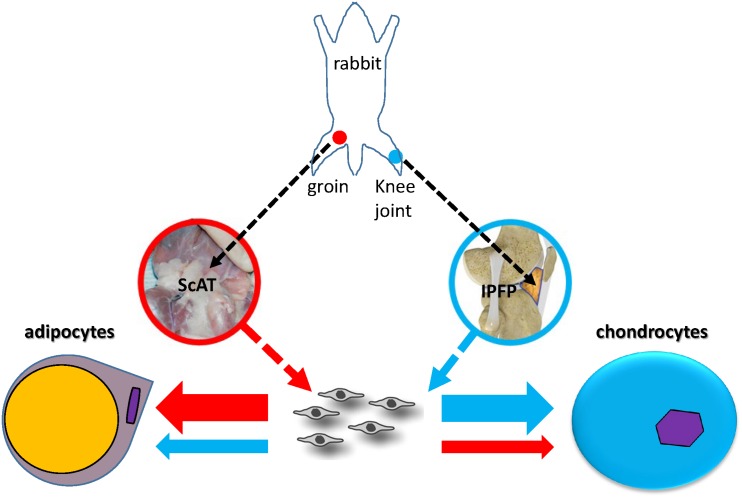

## Introduction

Adult stem cells, found in varied tissues such as bone marrow, adipose, and synovium, have the capacity for proliferation and differentiation and are important cell sources for clinical therapy (Brown et al., 2019). However, adult stem cells from different sources have different capacities for proliferation and tissue-specific differentiation (Pizzute et al., 2015). This discrepancy even exists in a single tissue, for example, adipose tissue. Recent evidence indicates that, compared with subcutaneous adipose tissue (ScAT), infrapatellar fat pad (IPFP) is a unique adipose tissue with some distinct features (Sun et al., 2018). ScAT, a fat pad beneath the skin, is becoming an attractive stem cell source for tissue regeneration due to its ease of harvest in large quantities with a minimally invasive procedure (Chu et al., 2019). IPFP, located inside the knee joint and outside the synovial membrane, is becoming a promising stem cell source for cartilage engineering and regeneration (Sun et al., 2018).

In osteoarthritis, IPFP exhibits both anti-inflammatory and pro-inflammatory characteristics (Alegre-Aguarón et al., 2012). Increasing evidence indicates the superiority of IPFP-derived stem cells (IPFSCs) over subcutaneous adipose stem cells (ScASCs) in chondrogenic differentiation capacity (Alegre-Aguarón et al., 2012; Lopa et al., 2014); however, most reports are not donor-matched studies, leading to an uncertain conclusion unless large-scale samples are used. In this study, donor-matched IPFSCs and ScASCs isolated from four New Zealand white rabbits were used for comparison in cell proliferation and tri-lineage differentiation capacities. We hypothesized that adipose stem cells have a depot-dependent lineage preference. Transcriptome and proteome analyses were used to facilitate an in-depth understanding of site-dependent variations of lineage preference.

## Materials and Methods

### Cell Culture and Proliferation and Surface Phenotype Evaluation

Donor-matched inguinal ScAT and IPFP were obtained from four 4-month-old male New Zealand white rabbits. This project was approved by our Institutional Animal Care and Use Committee (IACUC). The harvested tissues were finely minced and digested using 0.1% collagenase P (Roche, Indianapolis, IN, United States) for 90 min at 37°C in a shaking water bath. The collected cells were cultured in growth medium containing alpha-minimum essential medium (αMEM), 10% fetal bovine serum, 100 U/mL penicillin, 100 μg/mL streptomycin, and 0.25 μg/mL fungizone (Invitrogen, Carlsbad, CA, United States) in a 37°C, 5% CO_2_, humidified incubator. After reaching 80% confluence, ScASCs and IPFSCs were detached and counted (*n* = 4) using a hemocytometer, then seeded on T175 flasks at a density of 3000/cm^2^ for expansion. The cells were counted for three passages after harvesting. Cell population doubling time (PD time) was then calculated based on the formula “PD time = T^∗^log (2) / (log (N_1_) – log (N_0_))”. T stands for incubation time, N_1_ stands for harvesting cell number, and N_0_ stands for seeding cell number.

Passage 1 ScASCs and IPFSCs from four donors were used to perform EdU (5-ethynyl-2′-deoxyuridine) cell proliferation assay (cat no. C1024; Invitrogen) according to manufacturer’s protocols. Briefly, the cells were seeded at a density of 3000/cm^2^, grew to 40% confluence, and then were incubated with 10 μM EdU solution for 3 h before detaching, fixing, permeabilizing, and staining the cells. EdU fluorescence was detected and analyzed by FACSCalibur (BD Biosciences, San Jose, CA, United States) using FCS Express 5 software (De Novo Software, Los Angeles, CA, United States).

A CD146 assay was performed on passage 1 ScASCs and IPFSCs from four donors. Briefly, 3 × 10^5^ cells (*n* = 4) were incubated with CD146 antibody conjugated with phycoerythrin (cat no. 12-1469-42; Thermo Fisher Scientific, Milford, MA, United States) in darkness for 30 min. The fluorescence was analyzed by FACSCalibur using FCS Express 5 software (De Novo Software, Los Angeles, CA, United States).

### Stemness and Senescence Gene Expression

Expanded cells were evaluated using real-time quantitative polymerase chain reaction (qPCR) for differences between ScASCs and IPFSCs in stemness and senescence-related gene expression. Total RNA was extracted from passage 1 cells of all four donors using TRIzol (Invitrogen). Then cDNA was synthesized from mRNA by reverse transcriptase using a High-Capacity cDNA Archive Kit (Thermo Fisher Scientific). Primers of stemness-related genes, senescence-related genes, and an endogenous reference gene (Table 1) were customized from Integrated DNA Technologies (IDT, Coralville, IA, United States) as Sybr green gene expression assay using their qPCR tool. qPCR was performed with iCycler iQ^TM^ Multicolor RT-PCR Detection and calculated by computer software (PerkinElmer, Wellesley, MA, United States).

**TABLE 1:** Primers for qPCR.

Gene name	Full name	TaqMan Assay ID or IDT primers
**Stemness-related genes**		
*NANOG*	Nanog homeobox	Forward: TCAACTGCGGAGATGAAGTG;
		Reverse: GTTTGCTGTGCTGTGTTCTG
*REX1*	ZFP42 zinc finger protein	Forward: AGCCCAGCAGGCAGAAATGGAA;
		Reverse: TGGTCAGTCTCACAGGGCACAT
*NES*	nestin	Forward: AGAATTCCCGGCTTCAGACA;
		Reverse: TCTTCAGAAAGGCTGGCACA
*SOX2*	SRY-box2	Forward: TGAAGGAGCACCCGGATTAT;
		Reverse: GCAGCGTGTACTTATCCTTCTT
**Senescence-related genes**		
*CDKN1A*	Cyclin dependent kinase inhibitor 1A	Forward: ACCTCTCAGGGTCGGAAA;
		Reverse: GGATTACGGCTTCCTCTTGG
*TP53*	Tumor protein 53	Forward: CGTGCGGAGTATTTGGATGA;
		Reverse: TGGATGGTGGTACAGTCAGA
**Chondrogenesis-related genes**		
*SOX9*	SRY-box 9	Oc04096872_m1
*COL2A1*	Collagen type II alpha 1 chain	Oc03396132_g1
*ACAN*	Aggrecan	Forward: CTACGGAGACAAGGATGAGTTC;
		Reverse: CGTAAAAGACCTCACCCTCCAT
*COL10A1*	Collagen type X alpha 1 chain	Oc04097225_s1
*MMP13*	Matrix metallopeptidase 13	Oc03396895_m1
**Adipogenesis-related genes**		
*ADIPOQ*	Adiponectin, C1Q and collagen domain containing	Oc03823307_s1
*PPARG*	Peroxisome proliferator activated receptor gamma	Oc03397329_m1
*LEP*	Leptin	Oc03395809_s1
*LPL*	Lipoprotein lipase	Forward: GCAAGACCTTCGTGGTGAT;
		Reverse: GTTGGAGTCTGGTTCTCTCTTG
**Osteogenesis-related genes**		
*BGLAP*	Bone gamma-carboxyglutamate protein	ARMFW6N
*SPP1*	Secreted phosphoprotein 1	Oc04096883_g1
*DCN*	Decorin	Oc03398037_m1
*SPARC*	Secreted protein acidic and cysteine rich	Oc03395844_m1
**Endogenous control gene**		
*GAPDH*	Glyceraldhyde-3-phosphate dehydrogenase	Forward: TTCCACGGCACGGTCAAGGC;
		Reverse: GGGCACCAGCATCACCCCAC

### Chondrogenic Induction and Evaluation

Passage 2 expanded cells (4 × 10^5^) were centrifuged at 300 *g* for 7 min in a 15-ml polypropylene tube to form a pellet. After 24 h incubation (day 0), the pellets were cultured with serum free medium containing high-glucose Dulbecco’s modified Eagle’s medium, 40 μg/mL proline, 0.1 μM ascorbic acid-2-phosphate, 100 nM dexamethasone, 1 × ITS premix (BD Biosciences),100 U/mL penicillin, 100 μg/mL streptomycin, 0.25 μg/mL fungizone, and 10 ng/mL recombinant human transforming growth factor beta3 (TGFβ3, PeproTech Inc., Rocky Hill, NJ, United States) in a 37°C, 5% CO_2_, humidified incubator for up to 18 days.

Total RNA from ScASCs and IPFSCs (*n* = 4) was extracted from pellets using an RNase-free pestle in TRIzol, and other qPCR procedures were the same as described above. Primers for chondrogenic-related genes and the endogenous control gene (Table 1) were customized as TaqMan^®^ gene expression assay from Applied Biosystems (Foster City, CA, United States). *ACAN* primer was customized as Sybr green gene expression assay from IDT (Table 1).

### Adipogenic Induction and Evaluation

When passage 2 cells grew to 90% confluence, they were cultured in adipogenic medium consisting of growth medium supplemented with 1 μM dexamethasone, 10 μM insulin (Biovendor, Asheville, NC, United States), 0.5 mM 3-isobutyl-1-methylxanthine, and 200 μM indomethacin in a 37°C, 5% CO_2_, humidified incubator for as long as 21 days. Primers for qPCR analysis (*n* = 4) of adipogenic-related genes (Table 1) were customized as TaqMan^®^ gene expression assay from Applied Biosystems. *LPL* primer was customized as Sybr green gene expression assay from IDT (Table 1).

Adipogenic induced cell samples were dissolved in lysis buffer with protease inhibitors. Total protein was quantified by a NanoDrop Spectrophotometer. Twenty micrograms of proteins (*n* = 4) were separated on a 12% polyacrylamide gel and transferred to nitrocellulose membrane (30 V, overnight at 4°C). Membranes were blocked with 5% non-fat milk in TBS (Tris-buffered saline) and probed with primary adiponectin (ADIPOQ) monoclonal antibody (cat no. MA1-054; Thermo Fisher Scientific) followed by secondary antibody conjugated with HRP (horseradish peroxidase) (cat no. RK244131; Thermo Fisher Scientific). Next, a chemiluminescence kit (GE Healthcare, Chicago, IL, United States) was used for developing; the blot was imaged using a GE gel documentation. Anti-β actin antibody (Invitrogen) was used to normalize the loading amounts.

### Osteogenic Induction and Evaluation

When passage 2 cells grew to 90% confluence, they were cultured with osteogenic medium consisting of growth medium supplemented with 0.01 μM dexamethasone, 50 mg/L ascorbic acid-2-phosphate, and 10 mM β-glycerophosphate in a 37°C, 5% CO_2_, humidified incubator for as long as 21 days. Primers for qPCR analysis (*n* = 4) of osteogenic-related genes (Table 1) were customized as TaqMan^®^ gene expression assays from Applied Biosystems.

Protein extracts were assayed for osteocalcin using Osteocalcin (OCN) assay kit (cat no. MBS2019743; MyBioSource, San Diego, CA, United States) following manufacturer’s instructions. Briefly, protein samples (*n* = 4) were added to wells with pre-coated osteocalcin antibody and incubated at room temperature for 1 h. Upon incubation, 100 uL of detection reagent A was added to the microwells and incubated at 37°C for 1 h. Microwells were washed and incubated with 100 μL of detection reagent B at 37°C for 30 min. Upon washing, 90 μL of 3,3’,5,5’- tetramethylbenzidine substrate solution was added and the sample was incubated at 37°C for 30 min. The reaction was stopped by the addition of 50 μL of stop solution. This enzyme-linked immunosorbent assay (ELISA) was quantitatively measured using a microplate reader at 450 nm.

### RNA-Seq Analysis

The total RNA samples collected from donor-matched passage 1 IPFSCs (*n* = 4) and ScASCs (*n* = 4) underwent an initial quality control (QC) check. When samples were deemed high quality [RIN (RNA Integrity Number) > 8], a Next Generation Library was built followed by a final QC check, which involved Qubit quantification and size estimation on the Agilent Bioanalyzer using a High Sensitivity DNA chip. The completed libraries were sequenced on their HiSeq 2500 (Illumina, Inc., San Diego, CA, United States). FastQC^1^ and multiQC (Ewels et al., 2016) were used to confirm that the read quality was good and no trimming was required. We mapped the pair-end RNA-Seq reads to the reference genome of Oryctolagus cuniculus (OryCun2.0) from Ensembl (Zerbino et al., 2018) using the Subread aligner (Liao et al., 2013). The number of read counts corresponding to gene transcripts annotated by Ensembl (OryCun2.0.98) were summarized by the featureCounts function from the Rsubread package (Liao et al., 2019). Gene expression levels were quantified by Reads Per Kilobase of transcript, per Million mapped reads (RPKM) with in-house script. We applied Edger3 (McCarthy et al., 2012) to call differentially expressed genes with the following criteria: fold change (FC) > 2, false discovery rate (FDR) < 0.05, and an average expression (RPKM) higher than one in at least one condition.

### Proteomics Analysis of Donor-Matched Cells and Their Deposited Matrices

Donor-matched passage 1 IPFSCs (*n* = 4; C-IPFSC) and ScASCs (*n* = 4; C-ScASC) as well as the decellularized extracellular matrices (dECM) deposited by passage 1 IPFSCs (*n* = 4; E-IPFSC) and ScASCs (*n* = 4; E-ScASC) were used for this analysis. dECM was prepared following our previous protocol (Li and Pei, 2018). Briefly, 0.2% gelatin solution was used to coat a tissue culture plastic (TCP) flask for 1 h, followed by treatment with 1% glutaraldehyde and 1M ethanolamine for 30 min each. Both cells were cultured in the above-treated TCP flasks using growth medium until 100% confluence followed by supplementation with 250 μM L-ascorbic acid phosphate magnesium salt for 10 days and then treatment with Extraction Buffer (0.5% Triton X-100 and 20 mM NH_4_OH in phosphate buffered saline) (Pizzute et al., 2016). Cell lysates (50 μg) were diluted in 8 M urea in 100 mM ammonium bicarbonate (ABC) pH 8.5 for digestion according to the Filter Aided Sample Preparation (FASP) protocol. dECMs were processed as previously described (Barrett et al., 2017). Briefly, samples were lyophilized, and digested with 100 mM cyanogen bromide (CNBr). The resultant digest was solubilized in 8 M urea and FASP digested with sequencing grade trypsin (Wiśniewski et al., 2009). Liquid chromatography tandem mass spectrometry (LC-MS/MS) was performed on an Eksigent 2D nano LC coupled to an LTQ-Orbitrap Velos mass spectrometer. MS acquisition parameters were detailed previously (Hill et al., 2015). Raw files were directly loaded into Proteome Discoverer 2.2 3 (Thermo Fisher Scientific) and searched against mouse SwissProt database using an in-house Mascot^TM^ server (Version 2.5, Matrix Science). Mass tolerances were +/- 10ppm for MS peaks, and +/- 0.6 Da for MS/MS fragment ions. CNBr/Trypsin specificity was used allowing for 1 missed cleavage. Met oxidation, proline hydroxylation, protein N-terminal acetylation, and peptide N-terminal pyroglutamic acid formation were allowed for variable modifications while carbamidomethyl of Cys was set as a fixed modification. Label Free Quantifications were done using the Minora Feature detector for peak intensity based abundance. Protein thresholds of 1% FDR were used to filter for high confidence identifications. Results were directly exported into Microsoft Excel. Statistical enumeration of the data was achieved through Metaboanalyst (v3.6) (Xia and Wishart, 2016) and pathway analysis was done using the Reactome (Fabregat et al., 2018).

### Statistical Analyses

For the data from flow analysis, ELISA, and qPCR, results are presented as the mean and the standard deviation of the mean; the *t*-test was used to assess data between two groups. All statistical analyses were performed with SPSS 13.0 statistical software (SPSS, Inc., Chicago, IL, United States). *p* < 0.05 was considered statistically significant.

## Results

### Stem Cell Proliferation and Surface Marker Expression

**FIGURE 1:**
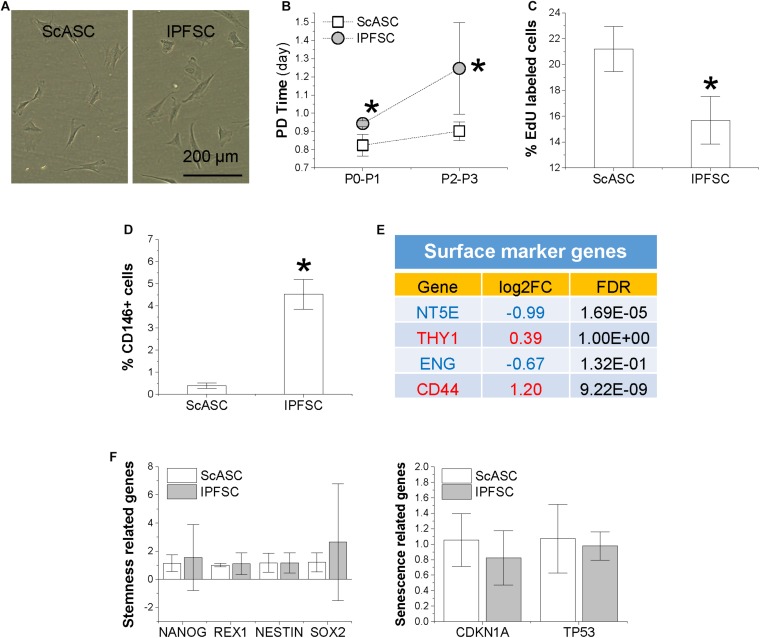
Comparison of proliferation capacity between ScASCs (*n* = 4) and donor-matched IPFSCs (*n* = 4) in **(A)** cell morphology; **(B)** cell population doubling (PD) time calculated by cell counting; **(C)** relative EdU incorporation; **(D)** CD146 surface marker expression level evaluated by flow cytometry analysis; **(E)** other surface marker gene expression levels evaluated by RNA-seq, in which a positive value in log2FC means higher expression in IPFSCs (in red) and a negative value means higher expression in ScASCs (in blue); and **(F)** stemness-related genes *NANOG*, *REX1*, *NESTIN*, and *SOX2* and senescence-related genes *CDKN1A* and *TP53* measured by qPCR. Data are shown as bar charts. * indicates a significant difference compared to the corresponding control group (*p* < 0.05).

ScASCs and donor-matched IPFSCs showed similar fibroblast-like morphology (Figure 1A). Population doubling (PD) time of ScASCs was significantly shorter than IPFSCs (Figure 1B) and relative EdU incorporation of ScASCs was higher than IPFSCs (Figure 1C), indicating that ScASCs were superior to donor-matched IPFSCs in cell proliferation. Surface phenotype assay showed that CD146 expression was negligible in ScASCs with an average of 0.39% (*n* = 4) while in donor-matched IPFSCs, the average CD146 expression was 4.52% (*n* = 4), significantly higher than ScASCs (Figure 1D). Due to the commercial unavailability of surface marker antibodies targeting rabbit CD44, CD73, CD90, and CD105, RNA-seq was used to measure these surface marker expressions at mRNA levels including *CD44* (Log2FC from IPFSCs to ScASCs was 1.20, *FDR = 9.22E-09*), *NT5E* (5′-Nucleotidase Ecto for CD73) (Log2FC from IPFSCs to ScASCs was −0.99, *FDR = 1.69E-05*), *THY1* (Thy-1 Cell Surface Antigen for CD90) (Log2FC from IPFSCs to ScASCs was 0.39, *FDR = 1.00E+00*), and *ENG* (Endoglin for CD105) (Log2FC from IPFSCs to ScASCs was −0.67, *FDR = 1.32E-01*) (Figure 1E). Interestingly, qPCR data showed that there were no significant differences in stemness-related genes *NANOG*, *REX1*, *NES*, and *SOX2*, and senescence-related genes *CDKN1A* and *TP53* in donor-matched adipose stem cells (Figure 1F).

### Adaptation of Transcription Factors During Differentiation Induction

**FIGURE 2:**
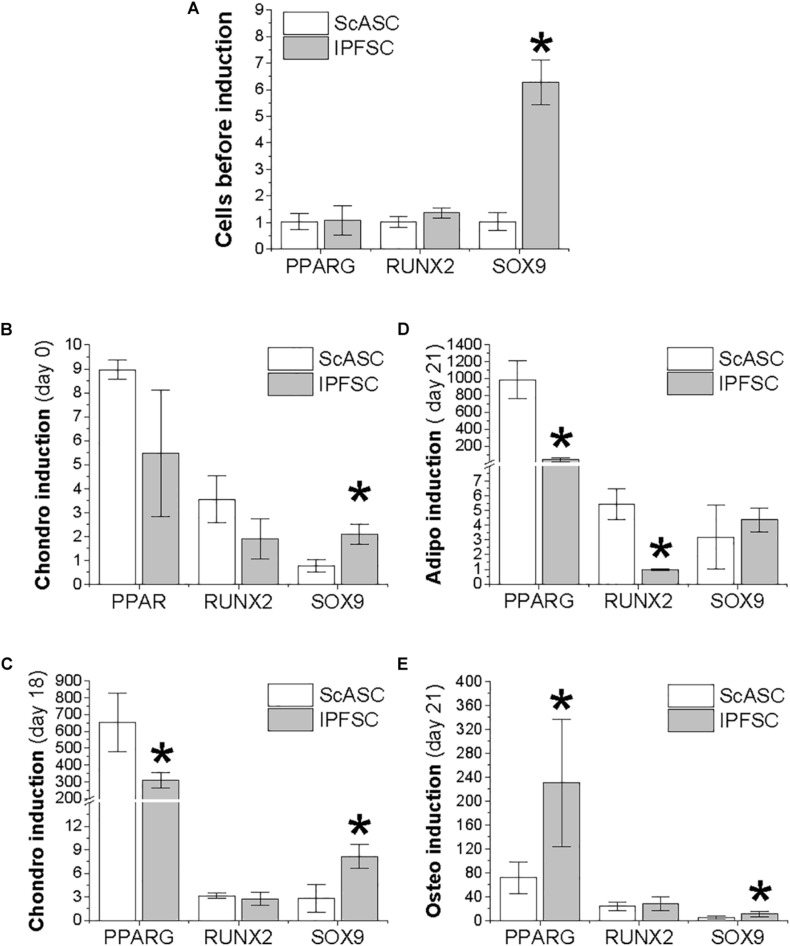
Expression of transcription factors in ScASCs (*n* = 4) and donor-matched IPFSCs (*n* = 4) measured by qPCR. *SOX9*, *PPARG*, and *RUNX2* were used to represent chondrogenesis, adipogenesis, and osteogenesis, respectively. Expanded cells were evaluated before induction **(A)** and after induction **(B–E)**: **(B)** 24 h after centrifugation to form a pellet but before chondrogenic induction; **(C)** 18 days after chondrogenic induction; **(D)** 21 days after adipogenic induction; and **(E)** 21 days after osteogenic induction. Data are shown as bar charts. Expression of each target gene in undifferentiated IPFSCs **(A)** and differentiated ScASCs/IPFSCs **(B–E)** is plotted against undifferentiated ScASCs **(A)**, which is set as “1”. * indicates a significant difference compared to the corresponding control group (*p* < 0.05).

To determine the adaptation of transcription factors in ScASCs and donor-matched IPFSCs, qPCR was used to evaluate *SOX9* for chondrogenesis, *PPARG* for adipogenesis, and *RUNX2* for osteogenesis during differentiation induction. Before induction, only *SOX9* expression was significantly higher in IPFSCs compared with ScASCs (Figure 2A). After an 18-day chondrogenic induction, *SOX9* expression was maintained at a higher level in IPFSCs similar to day 0 while *PPARG* expression was significantly lower than ScASCs despite a remarkable increase of *PPARG* in both cells (Figures 2B,C). After 21-day adipogenic induction, the difference in *SOX9* expression between groups was diminished; interestingly, both *PPARG* and *RUNX2* expression in IPFSCs were significantly lower than donor-matched ScASCs (Figure 2D). After a 21-day osteogenic induction, the difference in *SOX9* expression decreased but IPFSCs still had a higher level than ScASCs; intriguingly, IPFSCs exhibited a higher expression level in *PPARG* than ScASCs (Figure 2E).

### Development of Differentiation Factors During Induction

**FIGURE 3:**
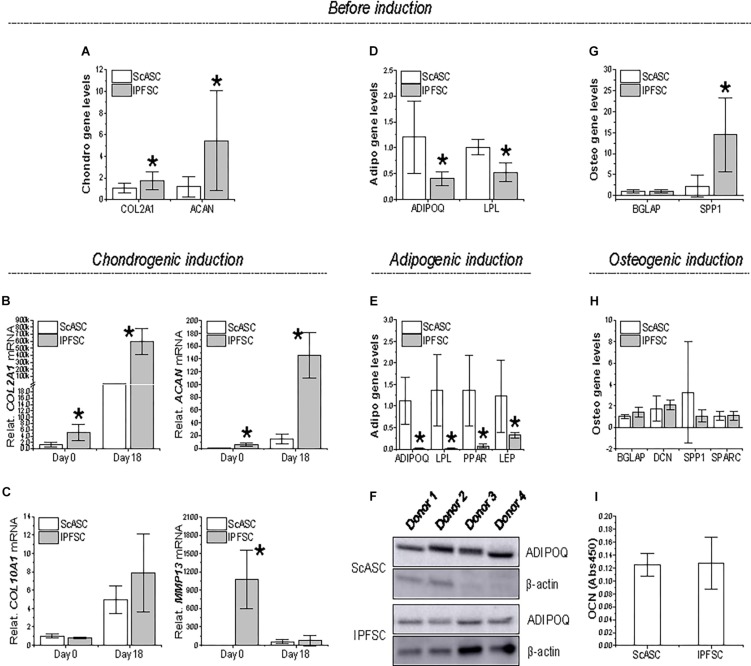
Expression of differentiation genes in ScASCs (*n* = 4) and donor-matched IPFSCs (*n* = 4) before and after induction. For chondrogenic differentiation, typical markers, *COL2A1* and *ACAN*, were evaluated using qPCR before **(A)** and after chondrogenic induction **(B)**. Hypertrophic markers *COL10A1* and *MMP13* were also measured after chondrogenic induction **(C)**. For adipogenic differentiation, typical markers, *ADIPOQ* and *LPL*, were evaluated using qPCR before **(D)** and after adipogenic induction **(E)**. *LEP* expression was also measured after induction **(E)**. Adiponectin (ADIPOQ) expression was confirmed using Western blot analysis with β-actin as an internal control **(F)**. For osteogenic differentiation, typical markers, *BGLAP* and *SPP1*, were evaluated using qPCR before **(G)** and after osteogenic induction **(H)**. Expression of *DCN* and *SPARC* were also measured after induction **(H)**. Osteocalcin (OCN) expression was confirmed using ELISA analysis **(I)**. Expression of each target gene in IPFSCs is plotted against the corresponding ScASCs, which is set as “1”, but for those in chondrogenic induction **(B/C)**, expression in day 0 is plotted as “1”. Data are shown as bar charts. * indicates a significant difference compared to the corresponding control group (*p* < 0.05).

To further explore the differentiation preference of donor-matched adipose stem cells, qPCR was used to evaluate *COL2A1* and *ACAN* as markers of chondrogenesis, *ADIPOQ* and *LPL* for adipogenesis, and *BGLAP* and *SPP1* for osteogenesis during induction. Before induction, IPFSCs had significantly higher expression of *COL2A1* and *ACAN* than ScASCs (Figure 3A), in line with *SOX9* expression (Figure 2A). The advantageous expression of *COL2A1* and *ACAN* was further strengthened during chondrogenic induction (Figure 3B). No significant difference of *COL10A1* expression was observed between groups; however, we found that *MMP13* expression was more than 1000 times higher in IPFSCs compared with ScASCs before induction, which became comparable between groups after chondrogenic induction (Figure 3C).

Before induction, ScASCs exhibited higher expression of *ADIPOQ* and *LPL* than IPFSCs (Figure 3D), despite no significant difference in transcription factor *PPARG* expression (Figure 2A). The dominant expression of *ADIPOQ* and *LPL* became dramatically increased during adipogenic induction along with differentiation gene *LEP* and transcription factor *PPARG* expression (Figure 3E). Moreover, higher expression of adiponectin (ADIPOQ) in ScASCs than donor-matched IPFSCs was also confirmed in Western blot analysis (Figure 3F).

For osteogenic differentiation, despite no difference in *BGLAP* expression before induction, IPFSCs displayed a higher expression of *SPP1* than ScASCs (Figure 3G), which became comparable after 21-day osteogenic induction along with other differentiation genes *BGLAP*, *DCN*, and *SPARC* expression (Figure 3H). Furthermore, comparable expression of OCN between ScASCs and IPFSCs was also confirmed in ELISA analysis (Figure 3I).

### Transcriptome Difference Between ScASCs and Donor-Matched IPFSCs

**FIGURE 4:**
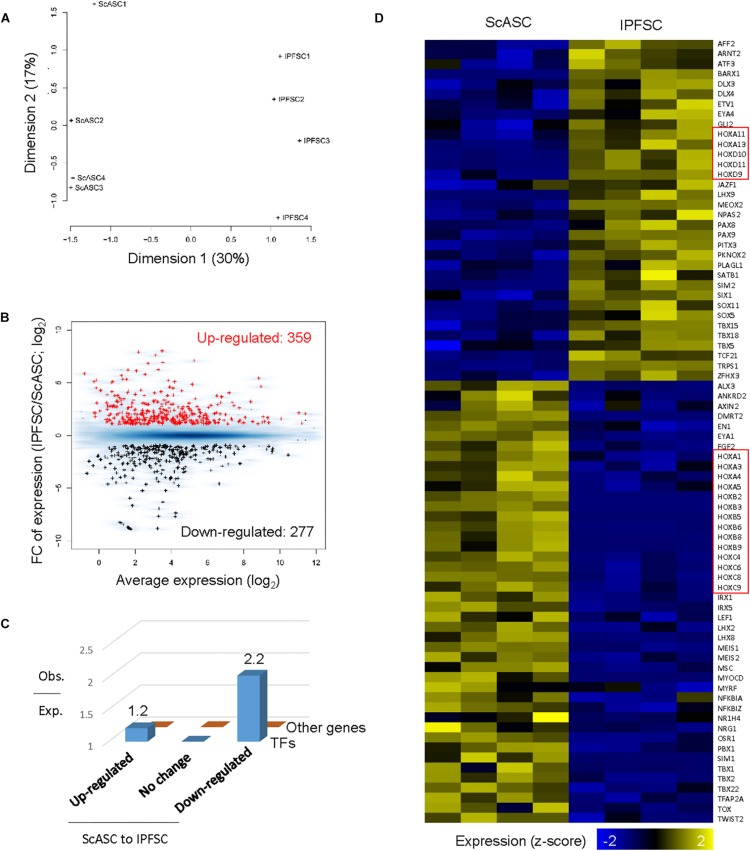
Differentially expressed gene analysis of ScASCs and donor-matched IPFSCs from four rabbits (*n* = 4). **(A)** Principal coordinates analysis on gene expression for RNA-Seq samples from ScASCs and IPFSCs. **(B)** MA plot showing the average and fold change (FC) of gene expression for differentially expressed genes between ScASCs and IPFSCs. Red: genes upregulated from ScASCs to IPFSCs; Black: genes downregulated from ScASC to IPFSCs; Blue background: all expressed genes. **(C)** Bar plot showing the ratio of observed number to the expected number of genes for transcription factor genes and for other genes grouped based on their expression changes between ScASCs and IPFSCs. **(D)** Heat map showing the expression changes between ScASCs and IPFSCs for differentially expressed transcription factors. Genes sorted first by their expression changes and then by names. Red rectangles: Homeobox (HOX) genes.

We applied RNA-seq data analysis to explore the difference in gene expression levels between ScASCs and IPFSCs. Principal coordinate analysis on gene expression across all genes revealed that samples from ScASCs and IPFSCs were from two distinctive clusters on the first dimension (Figure 4A). By applying EdgeR (McCarthy et al., 2012) with stringent thresholds [FC > 2 and FDR < 0.05], we identified 359 and 277 protein-coding genes upregulated and downregulated from the ScASC samples to the IPFSC samples, respectively (Figure 4B). Intriguingly, differentially expressed genes were enriched in those encoding transcription factors (Figure 4C), including the Homeobox (HOX) gene family related to stem cell differentiation (Seifert et al., 2015; Figure 4D).

**FIGURE 5:**
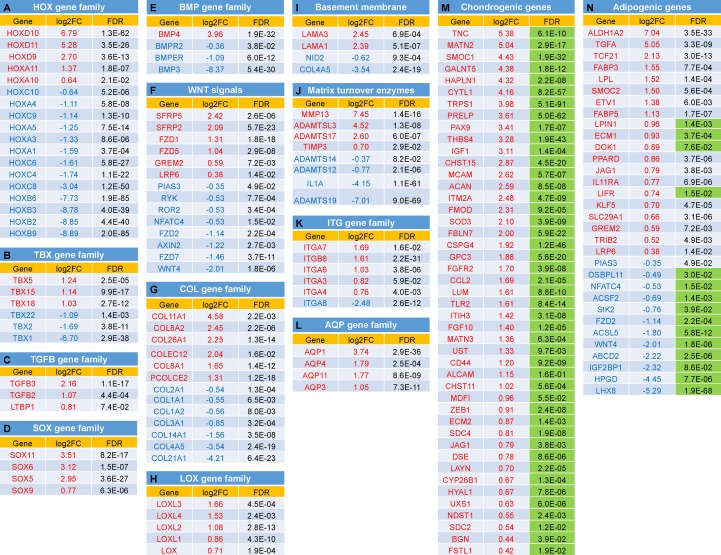
Expression changes between IPFSCs and ScASCs for selected groups of genes with function in chondrogenesis and/or adipogenesis: **(A)** Homeobox (HOX) gene family; **(B)** T (the brachyury gene)-box (TBX) gene family; **(C)** TGFβ (TGFB) gene family **(D)** SOX gene family; **(E)** Bone morphogenetic protein (BMP) gene family; **(F)** Wingless/integrase-1 (WNT) gene signals; **(G)** Collagen (COL) gene family; **(H)** Lysyl oxidase (LOX) gene family; **(I)** Genes coding for Basement membrane proteins; **(J)** Genes coding Matrix turnover enzymes; **(K)** Integrin (ITG) gene family; **(L)** Aquaporin (AQP) gene family; **(M)** Chondrogenic-related genes; and **(N)** Adipogenic-related genes. Green shaded FDR indicates favorable effect of the gene on chondrogenesis **(M)** or adipogenesis **(N)**. A positive value in log2FC means higher expression in IPFSCs (in red), and a negative value means higher expression in ScASCs (in blue). Orange shaded FDR: > 0.05.

We next examined the expression changes on particular groups of genes known to function in chondrogenesis and/or adipogenesis. The data were presented as Log2FC of expression and FDR. Functional annotation clustering was categorized (Figure 5) including (A) HOX gene family; (B) T (the brachyury gene)-box (TBX) gene family; (C) TGFβ (TGFB) gene family; (D) SOX gene family; (E) Bone morphogenetic protein (BMP) gene family; (F) Wingless/integrase-1 (WNT) gene signals; (G) Collagen (COL) gene family; (H) Lysyl oxidase (LOX) gene family; (I) Genes coding for Basement membrane proteins; (J) Genes coding Matrix turnover enzymes; (K) Integrin (ITG) gene family; (L) Aquaporin (AQP) gene family; (M) Chondrogenic-related genes; and (N) Adipogenic-related genes.

### Proteome Difference Between ScASCs and Donor-Matched IPFSCs

**FIGURE 6:**
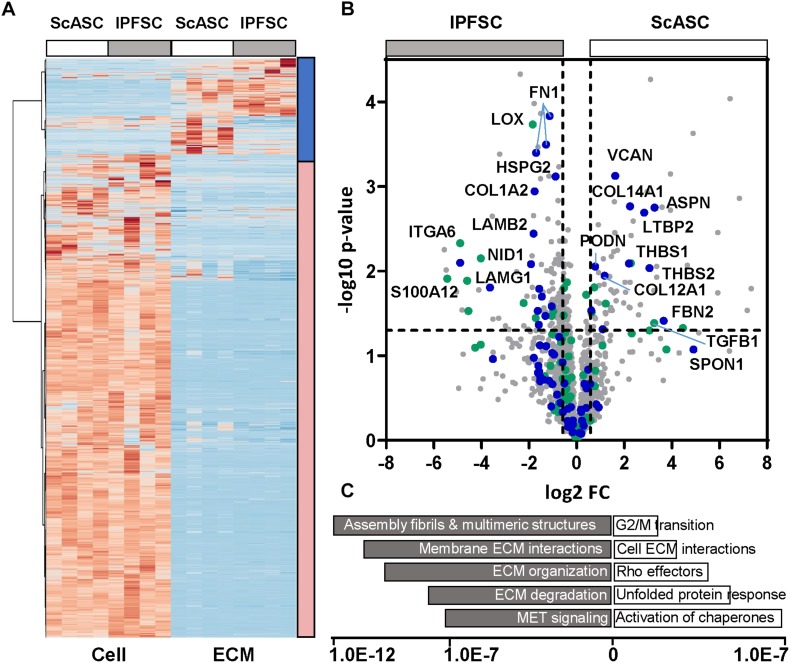
Proteomic results of cells and ECMs from ScASCs versus IPFSCs of four donor-matched rabbits. **(A)** Heatmap for global representation of protein differences (Z score used). The blue bar on the left indicates the region highly enriched in ECM components, the red bar indicates the region that contains primarily intracellular proteins. **(B)** Volcano plot with the *Y*-axis indicating -log10 *p*-value and the *X*-axis of the fold change (FC) in log2. Core ECM proteins are indicated in blue and Matrisome associated proteins are in green. **(C)** Gene enrichment analysis (top pathways, Reactome) is shown with the *X*-axis indicating the statistical significance based on false discovery rates using the Benjamani_hochberg method.

Both cells and deposited proteins were interrogated with bottom-up mass spectrometry-based proteomics to monitor differences in protein abundance (Figure 6A). The pathway analysis highlighted that the matrix proteins deposited by ScASCs mapped to more cell cycle regulators than traditional ECM proteins (Figures 6B,C), such as the uptick in fibril associated collagens with interrupted triple helices (FACIT collagens) including COL12A1 (FC = 2.26, *p* = 0.010) and COL14A1 (FC = 4.73, *p* = 0.002), Thrombospondin 1 (THBS1) (FC = 4.60, *p* = 0.008), THBS2 (FC = 8.37, *p* = 0.009), Asporin (ASPN, FC = 9.70, *p* = 0.002), Podocan (PODN, FC = 1.70, *p* = 0.009), and Versican (VCAN, FC = 3.10, *p* = 0.001). These proteins did not seem to be laying down as much ECM in general as those deposited by IPFSCs as shown by both the pathway analysis and the relatively lower amount of structural ECM.

In contrast, the matrix proteins enriched from IPFSCs include numerous basement membrane components (Figures 6B,C), such as nidogen 1 (NID1) (FC = 0.27, *p* = 0.008), heparin sulfate proteoglycan 2 (HSPG2)/perlecan (FC = 0.54, *p* = 0.001), laminin subunits including LAMA5 (FC = 0.10, *p* = 0.030), LAMB1 (FC = 0.40, *p* = 0.033), LAMB2 (FC = 0.28, *p* = 0.004), and LAMG1 (FC = 0.34, *p* = 0.005). Of relevance, and consistent with the mRNA level, the integrin A6 subunit (ITGA6) that forms a laminin receptor was also significantly increased (FC = 0.03, *p* = 0.005). In addition, various isoforms of fibronectin (FN) were significantly higher. Interestingly, compared to those secreted by ScASCs, collagen modifying enzymes, such as LOX and tissue transglutaminase (TG2, FC = 0.49, *p* = 0.050), accompanying fibrillar collagens, such as COL5A1 (FC = 0.36, *p* = 0.020) and COL5A3 (FC = 0.48, *p* = 0.030), were significantly upregulated in the matrix proteins deposited by IPFSCs. Consistent with the measured mRNA levels, LOX was significantly increased (FC = 0.27, *p* = 0.000) in matrix proteins deposited by IPFSCs, with similar trends in LOX-like 1 (LOXL1) (FC = 0.30, *p* = 0.036) and LOXL4 (FC = 0.48, *p* = 0.030).

## Discussion

Adult stem cells have the ability to differentiate into varied lineages. Increasing evidence indicates that adult stem cells from different tissues do not have equivalent ability toward a specific lineage differentiation (Jones and Pei, 2012; Pizzute et al., 2015). However, there is a lack of comparable studies using donor-matched adult stem cells and their matrix microenvironment. In this study, we found that, despite both deriving from adipose tissue, ScASCs and IPFSCs isolated from different depots of four donor-matched rabbits exhibited significant divergence in both proliferation and differentiation potential. Transcriptome and proteomics data provide robust evidence to support our hypothesis that there are site-dependent variations in lineage potential and preference of stem cells from adipose tissue.

Our proliferation data showed that ScASCs grew faster than donor-matched IPFSCs, which might result from higher levels of COL12A1, COL14A1, THBS1, THBS2, and VCAN expression in the matrix proteins deposited by ScASCs. These matrix proteins trend higher in tumor microenvironments and tissues to support proliferative or structurally rigid phenotypes (Wight et al., 2014; Subramanian and Schilling, 2015; Belotti et al., 2016). We also found that IPFSCs expressed significantly higher CD146 than donor-matched ScASCs, which was in line with the gene expression level in RNA-seq data (Log2FC from IPFSCs to ScASCs in CD146/*MCAM* was 2.62, *FDR* = 5.7E-07). Regardless of the role as a perivascular marker for adult stem cells (Crisan et al., 2008), a higher level of CD146 expression in adult stem cells might be associated with an advantage in chondrogenic differentiation (Hagmann et al., 2014; Su et al., 2015). Despite the failure to measure expression of surface markers CD90 and CD105 using flow cytometry on both ScASCs and IPFSCs (data not shown), our RNA-seq data indicated that the genes (*NT5E*, *THY1*, *ENG*, and *CD44*) encoding surface marker proteins (CD73, CD90, CD105, and CD44) were expressed in both cells with *NT5E* preferentially expressed in ScASCs and *CD44* advantageously expressed in IPFSCs. We also found no statistical difference in measured stemness-related genes (*NANOG*, *REX1*, *NESTIN*, and *SOX2*), indicating that these genes expressed in the donor-matched IPFSCs and ScASCs might not contribute to the discrepancy of a specific lineage differentiation found in this study.

Our differentiation data indicated that IPFSCs exhibited a significantly higher level of chondrogenic markers compared with donor-matched ScASCs. RNA-seq data showed that advantageous genes in IPFSCs can be categorized into several clusters based on functional annotation clustering. For example, the HOX gene, an evolutionarily highly conserved gene family, determines lineage specific differentiation of adult stem cells once tissue damage occurs (Seifert et al., 2015). In this study, HOXD genes, including *HOXD9-11* and *HOXA10/11*, were only dominantly expressed in IPFSCs over donor-matched ScASCs. HOXD genes, one of four grouped HOX clusters (HOXA, HOXB, HOXC, and HOXD) in vertebrates, are suggested to play a role in stem cell based chondrogenesis (Jung and Tsonis, 1998; González-Martín et al., 2014). *HOXA11* and *HOXD11* double mutant mice had shorter limb formation (Boulet and Capecchi, 2004). Compared with IPFSCs, however, donor-matched ScASCs exhibited robust expression of other HOX genes, such as *HOXA4*, *HOXA5*, *HOXC4*, and *HOXC8*, which are closely associated with adipogenesis rather than osteogenesis (Levi et al., 2010). An exception is *HOXC8* which does not inhibit but retards chondrogenesis, resulting in a boost of proliferating precursor cells and a negative influence on chondrogenesis (Yueh et al., 1998; Cormier et al., 2003).

Another group of conservative transcription factors, TBX family genes, also plays crucial roles throughout development. Our RNA-seq data showed that *TBX5*, *TBX15*, and *TBX18* were highly expressed in IPFSCs compared to donor-matched ScASCs. TBX5 was reported to activate WNT and fibroblast growth factor (FGF) signaling in upper limb development (Takeuchi et al., 2003). Overexpression of *TBX15* in 3T3-L1 preadipocytes impeded adipocyte differentiation and reduced triglyceride content (Gesta et al., 2011). *TBX15* and *TBX18* co-expressed in the limb mesenchyme indicate their functions during limb development (Tanaka and Tickle, 2004; Singh et al., 2005). Recently, *TBX18* was reported to co-express with *SOX9* in chondrogenic progenitors and differentiating chondrocytes (Haraguchi et al., 2015). In contrast, *TBX1* was reported to be preferentially expressed in the inducible pool of brown adipocytes (beige cells) present in white fat of the inguinal depot (Wu et al., 2012). The above findings indicate that two fat depots, ScAT and IPFP, exhibit distinct HOX and TBX conservative gene expression profiles that likely influence their differentiation preference.

The TGFβ superfamily is composed of two subfamilies; the TGFβ subfamily mainly includes TGFβs (1, 2, and 3) and the BMP subfamily mainly includes BMPs (2, 4-10) (Wang et al., 2014). A considerable amount of *in vitro* data indicate that TGFβ signaling pathways promote mesenchymal condensation (Barry et al., 2001; Tuli et al., 2003) and the early stages of chondrocyte differentiation, but restrain terminal hypertrophy (Mueller and Tuan, 2008; Shintani et al., 2013). In this study, we found that all TGFβ isotypes (*TGFB1*, *TGFB2*, and *TGFB3*) and latent-TGFB-binding protein 1 (*LTBP1*) were preferentially expressed in IPFSCs rather than donor-matched ScASCs. However, the protein level showed an opposite trend for TGFB1 and LTBP2, indicating that, while ScASCs have more available bound ligand, IPFSCs may have more free ligand, which needs to be further confirmed. This finding is in line with the advantageous expression in IPFSCs of the combination of *SOX5*, *SOX6*, and *SOX9* (the SOX trio) that supplies signals sufficient to induce permanent cartilage (Ikeda et al., 2004) and of *SOX11* that contributes to the regulation of growth/differentiation factor 5 (GDF5) in joint maintenance (Kan et al., 2013). Our data also showed that only *BMP4* exhibited a statistically higher level of expression in IPFSCs compared with donor-matched ScASCs. BMP4 plays an important role in retaining chondrogenic phenotype, through both increasing matrix production and suppressing the production of collagen type X (Reddi, 2001; Steinert et al., 2003). Another important BMP isotype in chondrogenesis, *BMP2*, was expressed at a higher level in IPFSCs than in donor-matched ScASCs despite no significant difference (*FDR* = 5.9E-01). Interestingly, *BMP3*, which was reported to inhibit bone formation by hindering BMP2 activity (Bahamonde and Lyons, 2001), was significantly expressed in ScASCs compared with donor-matched IPFSCs. These findings indicate that IPFSCs have an expression profile consistent with endochondral bone formation over donor-matched ScASCs. The above data support that TGFβ signaling cooperated with BMP signaling in stimulating articular cartilage regeneration (Wang et al., 2014).

Increasing evidence indicates that the canonical Wnt signaling pathway inhibits adipogenesis while the noncanonical Wnt pathway promotes adipogenesis by antagonizing the Wnt/β-catenin pathway (Christodoulides et al., 2009). In accordance with the above findings, we found that preferential expression favoring Wnt/β-catenin signaling genes such as SPARC-related modular calcium binding protein 2 (*SMOC2*) (Nie and Sage, 2009), low-density lipoprotein receptor-related protein 6 (*LRP6*) (Peröbner et al., 2012), and Gremlin 2 (*GREM2*) (Wu et al., 2015) in IFPSCs might be responsible for the inhibition of adipogenesis and the enhancement of chondrogenesis. In contrast, expression inhibiting Wnt/β-catenin signaling genes such as *WNT4* (Liu et al., 2016) and nuclear factor of activated T-cells 4 (*NFATC4*) (Yang et al., 2006) in ScASCs might be responsible for promoting adipogenesis and blocking chondrogenesis (Church et al., 2002).

Interestingly, we found FACIT collagen, such as *COL14A1* and *COL21A1*, was expressed at a higher level in ScASCs rather than IPFSCs. We also found that, despite collagen modifying enzymes detectable in both cells, IPFSCs exhibited significantly higher expression of LOX isoforms and tissue transglutaminase (Log2FC from IPFSCs to ScASCs for *TGM2* was 3.68, *FDR* = 4.9E-06) at both mRNA and protein levels than ScASCs, along with fibrillar collagens COL5A1 and COL5A3, which participate in the formation of a fibrillar collagen network and the regulation of fibrillogenesis (Imamura et al., 2000; Malfait et al., 2005; Lincoln et al., 2006). These findings indicate that IPFSCs are more suitable adipose stem cells for chondrogenesis compared to donor-matched ScASCs. During chondrogenesis, the contents of collagen type II and of non-reducible collagen cross-links were positively linked with tensile biomechanical properties and the functional integrity of cartilage tissues (Williamson et al., 2003). LOX family enzyme activities catalyze the eventual enzymatic conversion that is needed for the generation of biosynthetic collagen cross-links. For example, knockdown studies demonstrated that LOXL2 expression is required for mouse ATDC5 chondroprogenitors’ differentiation toward chondrocytes through regulation of *SNAIL* and *SOX9*, two important transcription factors for chondrogenesis (Iftikhar et al., 2011). Interestingly, hypoxia increased LOX gene expression, which promoted pyridinoline cross-links in engineered cartilage (Makris et al., 2013).

Regarding the matrix components, we found that IPFSCs deposited more basement membrane proteins compared to ScASCs, including *LAMA1*, *LAMA3*, and *HSPG2* at an mRNA level and LAMA5, LAMB1, LAMB2, LAMG1, NID1, and HSPG2 at a protein level. In contrast, *NID2* and *COL4A5* were significantly more highly expressed in ScASCs compared to donor-matched IPFSCs. Given the function of *HSPG2* encoding perlecan as the “factotum” in cartilage (Melrose et al., 2008) and the role of laminins in cartilage regeneration (Sun et al., 2017), *NID2* and *COL4A5* expression might be associated with adipogenic differentiation, which remains to be elucidated.

Interestingly, matrix turnover enzymes were uniquely preferentially expressed in IPFSCs or ScASCs. For example, matrix metallopeptidase 13 (MMP13), known as collagenase 3, was found exclusively in IPFSCs. The enzyme’s initial role is in tissue remodeling, especially during embryonic development, but it is also highly active in some pathological processes such as osteoarthritis and cancer (Inada et al., 2004; Tardif et al., 2004). In osteoarthritis, *MMP13* was highly expressed in chondrocytes and synovial cells responsible for the degradation of cartilage matrix (Inada et al., 2004; Davidson et al., 2006; van den Berg, 2011). However, little is known about *MMP13* expression in IPFP, either in healthy donors or in the pathological process. In this study, qPCR data showed that *MMP13* was expressed more than 1000-fold higher in IPFSCs than donor-matched ScASCs during cell condensation (day 0 pellet), in accordance with RNA-seq data of cell samples (Log2FC from IPFSCs to ScASCs in *MMP13* was 7.45, *FDR = 1.4E-16*). Interestingly, after an 18-day chondrogenic induction, IPFSCs exhibited a comparable expression of *MMP13* to donor-matched ScASCs. From the proteomics analysis, A disintegrin and metalloproteinase with thrombospondin motifs 5 (ADAMTS5) and ADAMTS1 were identified as significantly higher levels in IPFSCs versus a much higher level of ADAMTSL1 (ADAMTS like 1) in ScASCs. The protease profile is likely important for key processing events associated with the physiological role of IPFP in support of the remodeling of articular cartilage, which requires further investigation.

Integrin, a transmembrane receptor, facilitates cell-ECM adhesion. Interestingly, more integrins were advantageously expressed in IPFSCs including *ITGA3*, *ITGA4*, *ITGA6*, *ITGA7*, and *ITGB8* compared to *ITGA8*, which was superiorly expressed in donor-matched ScASCs. Another transmembrane protein, aquaporin, called the “the plumbing system for cells”, was found to serve as a water channel to rapidly move water through cells. Chondrocytes from articular cartilage are unique due to their sensitivity to water transfer and ionic and osmotic adjustments from the extracellular environment and are responsible for the creation of synovial fluid. A recent report showed that the expression of AQP1 and AQP3 were upregulated during chondrogenic induction of human adipose stem cells, indicative of physiological modification of functionally mature chondrocytes to the local environment (Graziano et al., 2018). This finding was consistent with our data, in which all statistically significant AQP gene expression was advantageously found in IPFSCs rather than donor-matched ScASCs, not only *AQP1* and *AQP3* but also *AQP4* and *AQP11*.

Furthermore, we found that important clusters associated with cartilage matrix were preferentially expressed in IPFSCs over donor-matched ScASCs. For instance, one of the clusters is glycosaminoglycan (GAG) metabolic and biosynthetic process, including N-Deacetylase And N-Sulfotransferase 1 (*NDST1*), Polypeptide N-Acetylgalactosaminyltransferase 5 (*GALNT5*), Biglycan (*BGN*), Carbohydrate Sulfotransferase 11 (*CHST11*), Dermatan-sulfate epimerase (*DSE*), Hyaluronidase-1 (*HYAL1*), and Inter-alpha-trypsin inhibitor heavy chain H3 (*ITIH3*), along with GAG binding function cluster, genes of *CD44*, *ACAN*, *BGN*, *BMP4*, Chemokine (C-C motif) ligand 2 (*CCL2*), Extracellular matrix protein 2 (*ECM2*), *FGF10*, *FGFR2*, Fibulin 7 (*FBLN7*), Follistatin-like 1 (*FSTL1*), Hyaluronan and proteoglycan link protein 1 (*HAPLN1*), Layilin (*LAYN*), *LPL*, procollagen C-endopeptidase enhancer 2 (*PCOLCE2*), Proline/arginine-rich end leucine-rich repeat protein (*PRELP*), Superoxide dismutase 3 (*SOD3*), *THBS4*, and Toll-like receoptor 2 (*TLR2*). The CD44 receptor transduces signals on chondrocytes, which is an important mediator of cell-matrix interactions, especially the homeostasis maintained by hyaluronan-CD44 interactions (Knudson and Loeser, 2002). Another crucial cluster is chondroitin sulfate proteoglycan and its metabolic process of which the advantageously expressed genes in IPFSCs include *ACAN*, *BGN*, Fibromodulin (*FMOD*), *HSPG2*, Syndecan 2 (*SDC2*), *SDC4*, *IGFI*, Cytokine-like 1 (*CYTL1*), *CHST11*, *CHST15*, Chondroitin sulfate N-acetylgalactosaminyltransferase 2 (*CSGALNACT2*), *DSE*, and Uronyl 2-sulfotransferase (*UST*). *IGFI* encodes insulin-like growth factor-I, which is the predominant anabolic growth factor and decreases matrix catabolism for articular cartilage (Jenniskens et al., 2006). Apparently, IPFSCs naturally express abundant genes involved in the chondrogenesis pathway by modulating cartilage development and cartilage ECM while ScASCs show no significant correlation with chondrogenesis.

Both ScASCs and donor-matched IPFSCs expressed plenty of genes related to lipid and fatty acid in their biosynthetic, catabolic, binding, and homeostasis processes. Among these differentially expressed genes, none of them showed a distinct tendency toward the brown adipose tissue due to either nondetectable expression of Uncoupling protein-1 (*UCP1*), Peroxisome proliferator-activated receptor-γ coactivator 1-α (*PGC1A*), and Forkhead box protein C2 (*FOXC2*), or comparable expression of Cell death inducing DFFA-like effector A (*CIDEA*) (Log2FC from IPFSCs to ScASCs was −1.45, *FDR* = 7.1E-01), a core set of brown adipose genes (Harms and Seale, 2013). Given the preferential expression of *PPARG*, a master regulator of adipogenesis (Kawai and Rosen, 2010), in ScASCs, most privileged associated genes favored adipogenesis, including but not limited to ATP Binding Cassette Subfamily G Member 1 (*ABCG1*) (Frisdal et al., 2015), Fibrillin-1 (*FBN1*) (Davis et al., 2016), *NFATC4* (Yang et al., 2006), Oxysterol-binding protein-related protein 11 (*OSBPL11*) (Zhou et al., 2012), Paternally expressed gene-10 (*PEG10*) (Hishida et al., 2007), Salt inducible kinase 2 (*SIK2*) (Henriksson et al., 2015), and *WNT4* (Christodoulides et al., 2009). Protein inhibitor of activated STAT 3 (*PIAS3*) did not favor adipogenesis (Deng et al., 2006). In contrast, excluding a few genes favoring adipogenesis, such as Docking protein 1 (*DOK1*) (Hosooka et al., 2008), *ECM1* (Challa et al., 2015), and Leukemia inhibitor factor receptor alpha (*LIFR*) (Aubert et al., 1999), most related genes inhibited adiogenic differentiation in IPFSCs, including but not limited to Fatty acid binding protein 4 (*FABP4*) (Garin-Shkolnik et al., 2014), *GREM2* (Wu et al., 2015), Jagged canonical NOTCH ligand 1 (*JAG1*) (Ugarte et al., 2009), *LRP6* (Peröbner et al., 2012), *SMOC2* (Nie and Sage, 2009), and Tribbles homolog 2 (*TRIB2*) (Naiki et al., 2007). The above findings indicate that, compared with ScASCs, donor-matched IPFSCs exhibit less potential to differentiate toward adipose tissue.

Our study did not find a significant difference in expression levels of osteogenic markers between ScASCs and donor-matched IPFSCs after induction. It has long been speculated that IPFSCs have a higher chondrogenic potential than other fat depots in the body (Sun et al., 2018), which might be due to the IPFP’s anatomic site that is closer to synovium and cartilage; it is regarded as a special form of fibro-adipose tissue (Macchi et al., 2016), or a continuation of synovium. IPFSCs may share some common characteristics with synovium-derived stem cells, a tissue-specific stem cell for chondrogenesis (Jones and Pei, 2012). A study comparing IPFSCs with near knee joint ScASCs from donor-matched osteoarthritis patients found that IPFSCs had a superior chondrogenic potential; ScASCs were superior in osteogenesis while both were similar in adipogenic capacity (Lopa et al., 2014). In this study with donor-matched normal adipose stem cells, we observed clear superiority of IPFSCs for chondrogenesis. However, we obtained divergent results on adipogenic and osteogenic potential. There were two possible explanations for the discrepancy; first, species differences between humans and rabbits can lead to considerable diversity, and secondly, Lopa et al. harvested IPFSCs from aged patients affected by osteoarthritis (Lopa et al., 2014) while we harvested from young healthy rabbits. The adult stem cell profile could be greatly affected by disease and aging (Li and Pei, 2012; Lynch and Pei, 2014).

Taken together, by donor-matched comparison and high throughput assay of transcriptome and proteomics, this study investigated the variance of depot-specific adult stem cells from adipose tissues. The findings might shed some light on the hypothesis that adipose stem cells have a depot-dependent lineage preference. Here, we have profiled RNA and protein levels to identify unique transcriptional programs, signaling components, and matrix environments that reveal novel insights into the higher chondrogenic potential of IPFSCs.

## Data Availability Statement

The RNA-Seq datasets generated for this study can be found in the GEO, under the accession number GSE142626, https://www.ncbi.nlm.nih.gov/geo/query/acc.cgi?acc=GSE142626. The data that support the findings of this study are available from the corresponding author upon reasonable request.

## Ethics Statement

The animal study was reviewed and approved by IACUC of West Virginia University, protocol number 1608003840.

## Author Contributions

TW contributed to the conception and design, collection and/assembly of data, data analysis and interpretation, manuscript writing, and final approval of the manuscript. RH and MD contributed to the collection and/assembly of data, data analysis and interpretation, manuscript writing, and final approval of the manuscript. AI, GH, LZ, and KH contributed to the data analysis and interpretation, manuscript writing, and final approval of the manuscript. MP contributed to the conception and design, financial support, data analysis and interpretation, manuscript writing, and final approval of the manuscript.

## Conflict of Interest

The authors declare that the research was conducted in the absence of any commercial or financial relationships that could be construed as a potential conflict of interest.

## Footnotes

1 https://www.bioinformatics.babraham.ac.uk/projects/fastqc/
